# Keratoconus Detection Based on a New Corneal Volumetric Analysis

**DOI:** 10.1038/s41598-017-16145-3

**Published:** 2017-11-20

**Authors:** Francisco Cavas-Martínez, Laurent Bataille, Daniel G. Fernández-Pacheco, Francisco J. F. Cañavate, Jorge L. Alio

**Affiliations:** 1 Technical University of Cartagena, Department of Graphical Expression, Cartagena, Spain; 2 Research and Development Department, Vissum Corporation Alicante, Alicante, Spain; 3 Division of Ophthalmology, Universidad Miguel Hernández, Alicante, Spain; 4 Keratoconus Unit of Vissum Corporation Alicante, Alicante, Spain; 5 Department of Refractive Surgery, Vissum Corporation Alicante, Alicante, Spain

## Abstract

There are numerous tomographic indices for the detection of keratoconus risk. When the indexes based on corneal volume are analyzed, two problems are presented: on the one hand, they are not very sensitive to the detection of incipient cases of keratoconus because they are not locally defined in the primary developmental region of the structural abnormalities; and on the other hand, they do not register the geometric decompensation driven by the asymmetry present during the disease progression. This work performed a morphogeometric modeling of the cornea by the aid of CAD tools and using raw topographic data (Sirius system, CSO, Firenze). For this method, four singular points present on the corneal surfaces were located and the following parameters based on corneal volume were calculated: VOL_mct_, defined by the points of minimal thickness; VOL_aap_, defined by the anterior corneal apex, and VOL_pap_, defined by the posterior corneal apex. The results demonstrate that a further reduction of corneal volume in keratoconus happens and significantly progresses along the disease severity level. The combination of optical and volumetric data, that collect the sensitivity of the asymmetry generated by the disease, allows an accurate detection of incipient cases and follow up of the disease progression.

## Introduction

Keratoconus is a corneal pathology that is characterized by a progressive deformation of the corneal curvature^1^, affecting the visual health of patients. The geometric characterization of the cornea in the pathology, which must include the final common pathway between the molecular, genetic and environmental factors that describe the origin and evolution of the pathology^2^, has been studied under a global approach^3^. However, the origin of the disease is local due to the development of structural abnormalities produced by an abnormal organization of the collagen fibers in a region of the stroma and by a loss in the anchoring capacity of the collagen fibrils in the Bowman layer^1,3^.

Keratoconus detection has been significantly optimized in the last years, evolving from simple topographic evaluations of the anterior corneal surface to accurate 3-D structural analyses of the whole cornea^4^. The consideration of the cornea as a solid with a specific volume has been suggested to be a potential and useful clinical tool for keratoconus detection and even for subclinical cases^5–13^. Likewise, the combination of pachymetric and volumetric data has demonstrated to provide a more efficient characterization of the corneal structure in keratoconus, with good levels of sensitivity and specificity for the detection of clinical and subclinical keratoconus^7,8,10,13^. Cui *et al*.^10^ found the existence of significant differences in corneal volume for the 3.0 mm and 5.0 mm central circles between healthy and subclinical keratoconus corneas. However, most of these studies have not been fully effective in detecting the disease because conceptually they consider the pathology in a global way, calculating volumes centered on the geometric center of the cornea, but not at the local point of manifestation of the asymmetry^14^. In this aspect, Ambrósio and colleagues^8^ considered the point of thinnest location as the center for computing the volumes and found statistically significant differences in the percentage of increase in volume between 3.5 mm and 7.0 mm diameters calculated from the tomographic data provided by the Pentacam system.

In a previous work, a morphogeometric modeling method allowing a characterization of the corneal structure as a solid and providing a comprehensive evaluation of the relationship between different sections of such solid model was proposed^5^. Specifically, new morphogeometric parameters were defined, including the area of the anterior and posterior corneal surface of the solid model generated, the area of the cornea within the sagittal plane passing through the Z axis and the highest point (apex) of the anterior corneal surface, the area of the cornea within the sagittal plane passing through the Z axis and the minimum thickness point of both corneal surfaces, or the average distance from the Z axis to the apex of both corneal surfaces^5^. From all the variables defined, the best diagnostic ability for keratoconus detection was found for anterior corneal surface area (area under the Receiver Operating Characteristic Curve, AUC: 0.847), posterior corneal surface area (AUC: 0.807), anterior apex deviation (AUC: 0.735) and posterior apex deviation (AUC: 0.891)^5^. The objective of this new study was to define, from singular points of the cornea and at local level, new volumetric parameters that collect the sensitivity of the asymmetry generated by the disease, combining them for the first time with optical data to evaluate their potential diagnostic ability for keratoconus detection with different levels of severity and to analyze its evolution along the progression of the disease.

## Patients and Methods

### Patients

This was an observational comparative study including 440 eyes of 440 patients ranging in age between 16 and 72 years old. Two groups of eyes were differentiated depending if the keratoconus disease was present or not: a control group, including 124 healthy eyes, and a keratoconus group, including 316 eyes with the diagnosis of keratoconus. Only one eye from each patient was randomly selected to be included in the study in order to avoid the interference in the analysis of the correlation parameters. The inclusion criterion for the control group was healthy eyes that did not meet the exclusion criteria, whereas the inclusion criteria for the keratoconus group was diagnosis of keratoconus based on standard guidelines^4,15^, including the presence of an asymmetric bowtie pattern in corneal topography, a value of 100 or higher of the KISA index, and at least one keratoconus sign on slit-lamp examination, such as stromal thining, conical protusion on the cornea at the apex, Fleischer ring, Vogt striae or anterior stromal scar. Exclusion criteria in both groups were previous ocular surgery or any other active ocular disease. The study was conducted at Vissum Corporation Alicante (centre affiliated with the Miguel Hernández University of Elche, Spain) and was approved by the ethics committee of this institution, being then performed in accordance with the ethical standards laid down in the 1964 Declaration of Helsinki (Seventh revision, October 2013, Fortaleza, Brasil). An informed consent was obtained from all subjects of the study.

### Examination protocol

All patients underwent a complete eye examination including the following tests: anamnesis, measurement of uncorrected (UDVA) and corrected distance visual acuity (CDVA), manifest refraction, slit-lamp biomicroscopy, and corneal analysis by the Sirius system (Costruzione Strumenti Oftalmici, Italy). According to the clinical data obtained in this examination, all cases were classified according to the Amsler-Krumeich grading system^4^.

### Morphogeometric modeling

The morphogeometric modeling used in the current study has been previously defined in detail and validated by our research group^5^. The procedure can be summarized in the following steps:

Step 1: Export of corneal topography files. All these files were exported in.csv format from the corneal topographer.

Step 2: Preparation of the point cloud. Exported CSV topography files provide raw data of the spatial points that conform both anterior and posterior corneal surfaces, indicating the coordinates of every scanned point in polar format (radii and semi-meridians), so an algorithm developed in Matlab software was used to convert data into Cartesian coordinates (X, Y, Z). For such purpose, every row of the CSV file was considered to represent a circle in the corneal map and every column a semi-meridian, providing a total of 256 points for each radius. Each i-th row sampled a map on a circle of i * 0.2 mm radius (0, 0.2, 0.4…6 mm), and each j-th column sampled a map on a semi-meridian in the direction of j * 360/256°, so each value of the matrix [i, j] represented the elevation of the point P (i * 0.2, j * 360/256°) in polar coordinates. However, due to the presence of extrinsic patient factors during the measurement process, such as the stability of the tear film, or an obstruction of the visual field by tabs or inadequate eyelid opening at the moment of the data collection, data provided by the Sirius device for determined points of the peripheral zones can be invalid, obtaining in these cases a value of −1000 in the corresponding matrix cells. Because of the presence of these erroneous values, a filtering process is performed to all the CSV files generated for each cornea, selecting for the study only those cases that contain in their first 21 rows (radii from 0 mm to 4 mm with respect to the normal corneal vertex) correct values (256 values for each row), discarding from the study any case in which an invalid −1000 value was detected within this range. This filtering process ensured that all data used for the generation of the point clouds was real and no interpolation was performed^5^.

Step 3: Geometric Surface Reconstruction. The point cloud representing the corneal geometry was imported into the surface reconstruction software Rhinoceros v5.0. The surface that best fits the point cloud was generated with the Rhinoceros’s patch surface function that tries to minimize the nominal distance between the 3D point cloud and the solution surface. The settings of the function were configured as follows: sample point spacing 256, surface span planes 255 for both u and v directions, and stiffness of the solution surface 10^–3^ (mm).

Step 4: Solid Modeling. The resulting surface was imported into the solid modeling software SolidWorks v2012. With this software, the solid model representing the custom and actual geometry of each cornea was generated.

Step 5: Definition and evaluation of the volumetric parameters.

### Volumetric parameters

**Figure 1:**
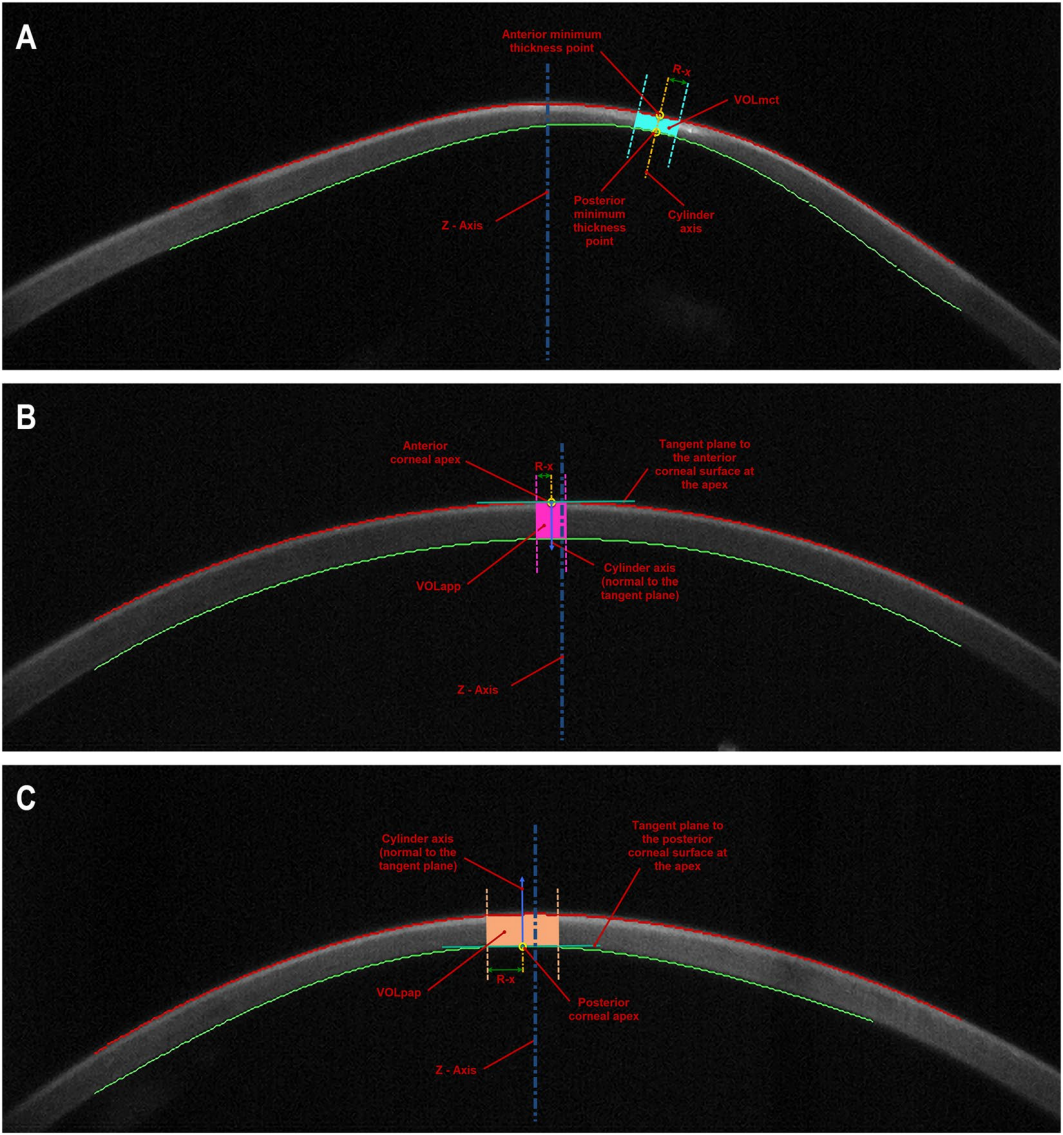
(**A**) Corneal volume (VOL_mct_) defined by the points of minimal thickness and a cylinder with radius R = 0.4 mm (Patient’s characteristics: KTCN IV, Age = 33, Sex = Female, Eye = Right, IOP = 10 mmHg, Central Thinkness = 372 µm), (**B**) Corneal volume (VOL_aap_) defined by the anterior corneal apex and a cylinder with radius R = 0.3 mm (Patient’s characteristics: KTCN I, Age = 40, Sex = Male, Eye = Right, IOP = 11 mmHg, Central Thinkness = 488 µm), (**C**) Corneal volume (VOL_pap_) defined by the posterior corneal apex and a cylinder with radius R = 0.7 mm (Patient’s characteristics: KTCN II, Age = 50, Sex = Female, Eye = Left, IOP = 12 mmHg, Central Thinkness = 471 µm).

From the solid model obtained for each cornea, the following volumetric variables were defined (Fig. 1):

- Corneal volume R-x (mm^3^) defined by the points of minimal thickness (VOL_mct_): volume contained in the intersection between the solid model of the cornea and a cylinder of revolution with radius x and its axis defined by the points of minimum corneal thickness of the anterior and posterior corneal surface. This volume was calculated for different radius values of the revolution cylinder, ranging from 0.1 to 1.5 mm.
- Corneal volume R-x (mm^3^) defined by the anterior corneal apex (VOL_aap_): volume contained in the intersection between the solid model of the cornea and a cylinder of revolution with radius x and its axis defined by a straight line perpendicular to the tangent plane to the anterior corneal surface at the apex. As VOL_mct_, this volume was also calculated for different radius values of the revolution cylinder, ranging from 0.1 to 1.5 mm.
- Corneal volume R-x (mm^3^) defined by the posterior corneal apex (VOL_pap_): volume contained in the intersection between the solid model of the cornea and a cylinder of revolution with radius x and its axis defined by a straight line perpendicular to the tangent plane to the posterior corneal surface at the apex. As VOL_mct_ and VOL_aap_, this volume was also calculated for different radius values of the revolution cylinder, ranging from 0.1 to 1.5 mm.

### Statistical analysis

SPSS statistics software package version 15.0 (IBM, Armonk, EEUU) was used for the statistical analysis. Normality of all data was checked by means of the Kolmogorov-Smirnov test. A comparison between healthy and keratoconus groups was performed with the unpaired Student t or Mann-Whitney U tests depending if the data samples were normally distributed or not. An additional analysis was performed to compare differences between groups according to keratoconus stages graded using the Amsler-Krumeich classification system. The one-way analysis of variance (ANOVA) was used for such purpose if variables were normally distributed, whereas the Kruskal-Wallis test was used if one or more variables were not normally distributed. The post-hoc comparative analysis for the ANOVA was performed with the Bonferroni test when the variances were homogeneous and the T2 Tamhane test when the variances were not homogeneous, while the Mann-Whitney tests with the Bonferroni’s adjustment was used for the post-hoc analysis of the Kruskal-Wallis test. Pearson and Spearman correlation coefficients were used to assess the correlation between anterior and posterior geometric parameters depending if the data samples were or not normally distributed. Differences were considered to be statistically significant when the associated p-value was <0.05.

A stepwise backward logistic regression was also performed to define the key parameters involved in the detection of keratoconus grade I as moderate and severe keratoconus can be easily detected by means of topographic and biomicroscopic analysis. Hosmer-Lemeshow adjustment was used to assess the overall goodness of fit of the model, and R^2^ Cox and Snell andR^2^ Nagelkerke were used to study the variance rate explained by the variables of the model. Finally, the efficacy of the model to detect keratoconus grade I was compared with that provided by the classifier of the topography system used for obtaining the measurements. This classifier is based on the use of different indices obtained from both anterior and posterior corneal surfaces, including symmetry index of front and back corneal curvature, best fit radius of the front corneal surface, Baiocchi Calossi Versaci front index (BCV(f)) and BCV back index (BCV(b)), root mean square of front and back corneal surface higher order aberrations, and thinnest corneal point^16^.

### Data availability

Data are available from the VISSUM Corporation Institutional Data Access/Ethics Committee for researchers who meet the criteria for access to confidential data.

### Ethical approval

All procedures performed in studies involving human participants were in accordance with the ethical standards of the institutional and/or national research committee and with the 1964 Helsinki declaration and its later amendments or comparable ethical standards.

## Results

A total of 124 healthy eyes of 124 patients (28.2%) (control group) and 316 keratoconus eyes of 316 patients (71.8%) (keratoconus group) were enrolled in the study. In the keratoconus group, the following subgroups were differentiated according to the stage of the disease following the Amsler-Krumeich grading system: grade I (223 eyes, 70.6%), grade II (57 eyes, 18.0%), grade III (9 eyes, 2.8%), and grade IV (27 eyes, 8.5%). Table 1 summarizes the main clinical variables characterizing the control and keratoconus group.

**Table 1:** Summary of the main clinical variables in control and keratoconus groups. Abbreviations: SD, standard deviation; D, diopter; UDVA, uncorrected distance visual acuity; CDVA, corrected distance visual acuity; MCT, minimum corneal thickness; CCT, central corneal thickness; RMS, root mean square; HOA, high order aberrations; SA, spherical aberration.

Mean (SD); Median (Range)	Control	Keratoconus	p-valor
*LogMAR UDVA*	0.63 (0.88); 0.40 (−0.08 to 3.00)	0.93 (0.87); 0.70 (−0.18 to 3.00)	0.004
*Sphere (D)*	−0.55 (3.76); 0.00 (−12.50 to 8.50)	−2.44 (4.45); −1.00 (−20.00 to 5.00)	<0.001
*Cylinder (D)*	−0.63 (0.75); −0.50 (−5.75 to 0.00)	−2.76 (2.38); −2.25 (−17.00 to 0.00)	<0.001
*Spherical equivalent (D)*	−0.87 (3.70); 0.00 (−12.88 to 8.12)	−3.76 (4.53); −2.38 (−21.75 to 4.00)	<0.001
*LogMAR CDVA*	0.00 (0.04); 0.00 (−0.08 to 0.22)	0.24 (0.42); 0.10 (−0.18 to 3.00)	<0.001
*MCT (µm)*	541.09 (32.0); 541.00 (480 to 634)	449.56 (66.09); 455.00 (231 to 602)	<0.001
*CCT (µm)*	544.33 (32.27); 544.50 (482 to 639)	468.38 (59.82); 475.00 (285 to 633)	<0.001
***Corneal aberrations:***			
*RMS HOA (µm)*	0.42 (0.11); 0.40 (0.24 to 0.76)	2.56 (2.24); 2.01 (0.32 to 13.84)	<0.001
*SA (µm)*	0.22 (0.06); 0.22 (0.08 to 0.42)	−0.18 (1.09); 0.13 (−7.85 to 1.32)	<0.001
*RMS coma (µm)*	0.28 (0.12); 0.26 (0.02 to 0.61)	2.06 (1.98); 1.50 (0.04 to 12.85)	<0.001
*Coma-like RMS (µm)*	0.33 (0.13); 0.32 (0.08 to 0.70)	2.31 (2.03); 1.86 (0.20 to 12.95)	<0.001
*Spherical-like RMS (µm)*	0.24 (0.06); 0.24 (0.11 to 0.48)	0.92 (1.07); 0.60 (0.15 to 8.29)	<0.001
***Corneal asphericity:***			
*4*.*5* *mm*	−0.09 (0.27); −0.07 (−0.65 to 0.84)	−0.50 (1.48); −0.44 (−7.42 to 4.10)	0.002
*8* *mm*	−0.25 (0.19); −0.25 (−0.78 to 0.13)	−0.80 (0.80); −0.63 (−3.00 to 2.82)	<0.001

### Comparison control vs. keratoconus group

**Figure 2:**
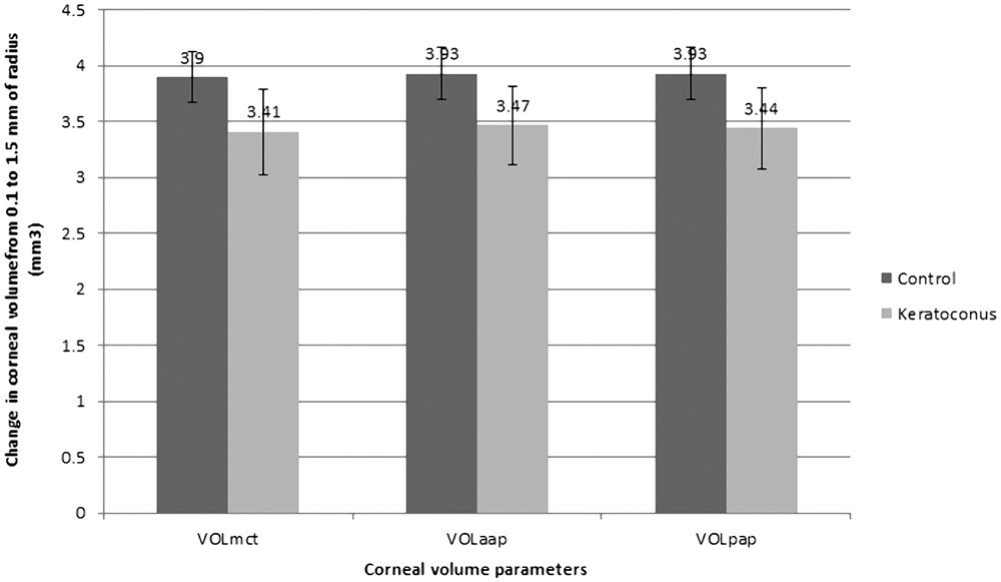
Change in corneal volume parameters from 0.1 to 1.5 mm of radius of the cylinder of revolution considered for their calculation. Abbreviations: VOL_mct_, corneal volume defined by the points of minimal thickness; VOL_aap_, corneal volume defined by the anterior corneal apex; VOL_pap_, corneal volume defined by the posterior corneal apex.

Table 2 summarizes the corneal volume outcomes obtained in the control and keratoconus group. As shown, significant differences were found among control and keratoconus groups in all corneal volume parameters calculated (p < 0.001). Specifically, in keratoconus group, significant lower values of VOL_mct_, VOL_aap_, and VOL_pap_ were found (p < 0.001). Likewise, significant differences were found between groups in the change of these corneal volumes from 0.1 to 1.5 mm of radius for the cylinder of revolution considered for their calculation (p < 0.001) (Fig. 2) (ΔVOL_mct_, ΔVOL_aap_, and ΔVOL_pap_), with the lower values in the keratoconus group.

**Table 2:** Summary of the corneal volume outcomes obtained in control and keratoconus groups.

Mean (SD); Median (Range)	Control	Keratoconus	p-valor
**VOL**_**mct**_ **(mm** ^**3**^ **)**			
*Radius 0*.*1 mm*	0.017 (0.001); 0.017 (0.015 to 0.020)	0.014 (0.002); 0.014 (0.010 to 0.020)	<0.001
*0*.*2 mm*	0.068 (0.004); 0.068 (0.060 to 0.080)	0.057 (0.008); 0.058 (0.020 to 0.080)	<0.001
*0*.*3 mm*	0.153 (0.009); 0.153 (0.140 to 0.180)	0.128 (0.018); 0.130 (0.060 to 0.170)	<0.001
*0*.*4 mm*	0.27 (0.02); 0.27 (0.24 to 0.32)	0.23 (0.03); 0.23 (0.10 to 0.31)	<0.001
*0*.*5 mm*	0.43 (0.03); 0.43 (0.38 to 0.50)	0.36 (0.05); 0.36 (0.16 to 0.48)	<0.001
*0*.*6 mm*	0.61 (0.04); 0.61 (0.54 to 0.72)	0.52 (0.07); 0.53 (0.24 to 0.69)	<0.001
*0*.*7 mm*	0.84 (0.05); 0.84 (0.74 to 0.98)	0.71 (0.09); 0.72 (0.33 to 0.94)	<0.001
*0*.*8 mm*	1.09 (0.06); 1.09 (0.97 to 1.28)	0.93 (0.12); 0.94 (0.44 to 1.24)	<0.001
*0*.*9 mm*	1.39 (0.09); 1.39 (1.00 to 1.63)	1.18 (0.15); 1.20 (0.58 to 1.57)	<0.001
*1*.*0 mm*	1.72 (0.11); 1.72 (1.52 to 2.01)	1.47 (0.18); 1.49 (0.72 to 1.94)	<0.001
*1*.*1 mm*	2.08 (0.12); 2.08 (1.85 to 2.44)	1.79 (0.22); 1.81 (0.90 to 2.36)	<0.001
*1*.*2 mm*	2.49 (0.15); 2.49 (2.20 to 2.91)	2.14 (0.25); 2.17 (1.09 to 2.81)	<0.001
*1*.*3 mm*	2.93 (0.17); 2.93 (2.60 to 3.42)	2.53 (0.30); 2.55 (1.31 to 3.31)	<0.001
*1*.*4 mm*	3.40 (0.20); 3.41 (3.00 to 3.98)	2.96 (0.33); 2.98 (1.56 to 3.86)	<0.001
*1*.*5 mm*	3.92 (0.23); 3.92 (3.48 to 4.58)	3.42 (0.38); 3.44 (1.84 to 4.44)	<0.001
**VOL**_**aap**_ **(mm** ^**3**^ **)**			
*Radius 0*.*1 mm*	0.017 (0.001); 0.017 (0.015 to 0.020)	0.015 (0.002); 0.015 (0.010 to 0.020)	<0.001
*0*.*2 mm*	0.068 (0.004); 0.068 (0.060 to 0.080)	0.059 (0.007); 0.060 (0.040 to 0.080)	<0.001
*0*.*3 mm*	0.154 (0.009); 0.154 (0.140 to 0.180)	0.133 (0.016); 0.135 (0.080 to 0.180)	<0.001
*0*.*4 mm*	0.27 (0.02); 0.27 (0.24 to 0.32)	0.24 (0.03); 0.24 (0.14 to 0.32)	<0.001
*0*.*5 mm*	0.43 (0.03); 0.43 (0.38 to 0.50)	0.37 (0.04); 0.38 (0.23 to 0.50)	<0.001
*0*.*6 mm*	0.62 (0.04); 0.62 (0.55 to 0.72)	0.54 (0.06); 0.54 (0.33 to 0.72)	<0.001
*0*.*7 mm*	0.84 (0.05); 0.84 (0.75 to 0.99)	0.73 (0.08); 0.74 (0.45 to 0.98)	<0.001
*0*.*8 mm*	1.10 (0.07); 1.10 (0.98 to 1.29)	0.96 (0.11); 0.97 (0.60 to 1.28)	<0.001
*0*.*9 mm*	1.40 (0.09); 1.40 (1.00 to 1.64)	1.22 (0.14); 1.23 (0.77 to 1.62)	<0.001
*1*.*0 mm*	1.74 (0.11); 1.73 (1.53 to 2.02)	1.51 (0.17); 1.52 (0.96 to 2.01)	<0.001
*1*.*1 mm*	2.10 (0.12); 2.10 (1.86 to 2.46)	1.84 (0.20); 1.85 (1.18 to 2.43)	<0.001
*1*.*2 mm*	2.50 (0.15); 2.50 (2.00 to 2.93)	2.19 (0.23); 2.21 (1.43 to 2.90)	<0.001
*1*.*3 mm*	2.95 (0.17); 2.95 (2.61 to 3.44)	2.59 (0.27); 2.60 (1.71 to 3.42)	<0.001
*1*.*4 mm*	3.43 (0.20); 3.42 (3.00 to 4.00)	3.02 (0.31); 3.03 (2.01 to 3.98)	<0.001
*1*.*5 mm*	3.95 (0.23); 3.95 (3.50 to 4.61)	3.48 (0.35); 3.49 (2.36 to 4.58)	<0.001
**VOL**_**pap**_ **(mm** ^**3**^ **)**			
*Radius 0*.*1 mm*	0.017 (0.001); 0.017 (0.015 to 0.020)	0.015 (0.002); 0.015 (0.010 to 0.020)	<0.001
*0*.*2 mm*	0.068 (0.004); 0.068 (0.060 to 0.080)	0.058 (0.008); 0.059 (0.030 to 0.080)	<0.001
*0*.*3 mm*	0.154 (0.009); 0.154 (0.140 to 0.180)	0.131 (0.017); 0.133 (0.060 to 0.180)	<0.001
*0*.*4 mm*	0.27 (0.02); 0.27 (0.24 to 0.32)	0.23 (0.03); 0.24 (0.12 to 0.31)	<0.001
*0*.*5 mm*	0.43 (0.03); 0.43 (0.38 to 0.50)	0.37 (0.05); 0.37 (0.19 to 0.49)	<0.001
*0*.*6 mm*	0.62 (0.04); 0.62 (0.55 to 0.72)	0.53 (0.07); 0.54 (0.27 to 0.70)	<0.001
*0*.*7 mm*	0.84 (0.05); 0.84 (0.75 to 0.99)	0.72 (0.09); 0.73 (0.37 to 0.96)	<0.001
*0*.*8 mm*	1.10 (0.07); 1.10 (0.98 to 1.29)	0.95 (0.11); 0.96 (0.50 to 1.25)	<0.001
*0*.*9 mm*	1.40 (0.09); 1.40 (1.00 to 1.64)	1.20 (0.14); 1.22 (0.64 to 1.58)	<0.001
*1*.*0 mm*	1.74 (0.11); 1.73 (1.53 to 2.02)	1.49 (0.18); 1.51 (0.80 to 1.96)	<0.001
*1*.*1 mm*	2.10 (0.12); 2.10 (1.86 to 2.45)	1.82 (0.21); 1.84 (0.99 to 2.38)	<0.001
*1*.*2 mm*	2.50 (0.15); 2.50 (2.00 to 2.92)	2.17 (0.24); 2.19 (1.20 to 2.84)	<0.001
*1*.*3 mm*	2.94 (0.17); 2.94 (2.61 to 3.44)	2.56 (0.28); 2.58 (1.43 to 3.35)	<0.001
*1*.*4 mm*	3.42 (0.20); 3.42 (3.00 to 4.00)	2.99 (0.32); 3.01 (1.70 to 3.89)	<0.001
*1*.*5 mm*	3.95 (0.23); 3.95 (3.50 to 4.60)	3.46 (0.36); 3.47 (2.00 to 4.49)	<0.001

Abbreviations: SD, standard deviation; VOL_mct_, corneal volume defined by the points of minimal thickness; VOL_aap_, corneal volume defined by the anterior corneal apex; VOL_pap_, corneal volume defined by the posterior corneal apex. These volumes were calculated for different radius values of the revolution cylinder, ranging from 0.1 to 1.5 mm.

Table 3 summarizes the outcomes obtained in the control group and keratoconus subgroups according to the stage of severity of the disease. Significant differences among keratoconus stages were found in ΔVOL_mct_, ΔVOL_aap_, and ΔVOL_pap_ (p < 0.001). Specifically, significant differences were found among all keratoconus subgroups (p ≤ 0.001), except for the comparison between eyes with keratoconus grade III and IV (p = 0.999).

**Table 3:** Summary of the outcomes obtained in control group and keratoconus subgroups according to the stage of severity of the disease.

Mean (SD) Median (Range)	Control (C)	Ktc grade I (KC1)	Ktc grade II (KC2)	Ktc grade III (KC3)	Ktc grade IV (KC4)	p-valor	Post-hoc analysis
ΔVOL_mct_ (mm^3^)	3.90 (0.23) 3.91 (3.47 to 4.56)	3.52 (0.32) 3.50 (1.83 to 4.43)	3.27 (0.34) 3.25 (2.00 to 3.90)	2.83 (0.31) 2.93 (2.32 to 3.20)	2.95 (0.29) 2.94 (2.35 to 3.43)	<0.001	C-KC1 <0.001 C-KC2 <0.001 C-KC3 <0.001 C-KC4 <0.001 KC2-KC3 0.001 KC2-KC3 <0.001 KC3-KC4 0.999
ΔVOL_aap_ (mm^3^)	3.93 (0.23) 3.93 (3.49 to 4.59)	3.57 (0.30) 3.56 (2.35 to 4.56)	3.34 (0.28) 3.33 (2.63 to 3.92)	2.91 (0.33) 3.00 (2.36 to 3.27)	3.04 (0.27) 3.02 (2.54 to 3.56)	<0.001	C-KC1 <0.001 C-KC2 <0.001 C-KC3 <0.001 C-KC4 <0.001 KC2-KC3 <0.001 KC2-KC3 <0.001 KC3-KC4 0.999
ΔVOL_pap_ (mm^3^)	3.93 (0.23) 3.93 (3.48 to 4.58)	3.55 (0.31) 3.54 (1.99 to 4.47)	3.32 (0.29) 3.30 (2.52 to 3.90)	2.87 (0.32) 2.95 (2.34 to 3.23)	2.99 (0.28) 2.99 (2.49 to 3.49)	<0.001	C-KC1 <0.001 C-KC2 <0.001 C-KC3 <0.001 C-KC4 <0.001 KC2-KC3 <0.001 KC2-KC3 <0.001 KC3-KC4 0.999

Abbreviations: SD, standard deviation; ΔVOL_mct_, change in corneal volume defined by the points of minimal thickness from 0.1 to 1.5 mm of radius for the cylinder of revolution considered for its calculation; ΔVOL_aap_, change in corneal volume defined by the anterior corneal apex from 0.1 to 1.5 mm of radius for the cylinder of revolution considered for its calculation; ΔVOL_pap_, change in corneal volume defined by the posterior corneal apex from 0.1 to 1.5 mm of radius for the cylinder of revolution considered for its calculation.

### Correlation between corneal volume parameters and clinical data

**Figure 3:**
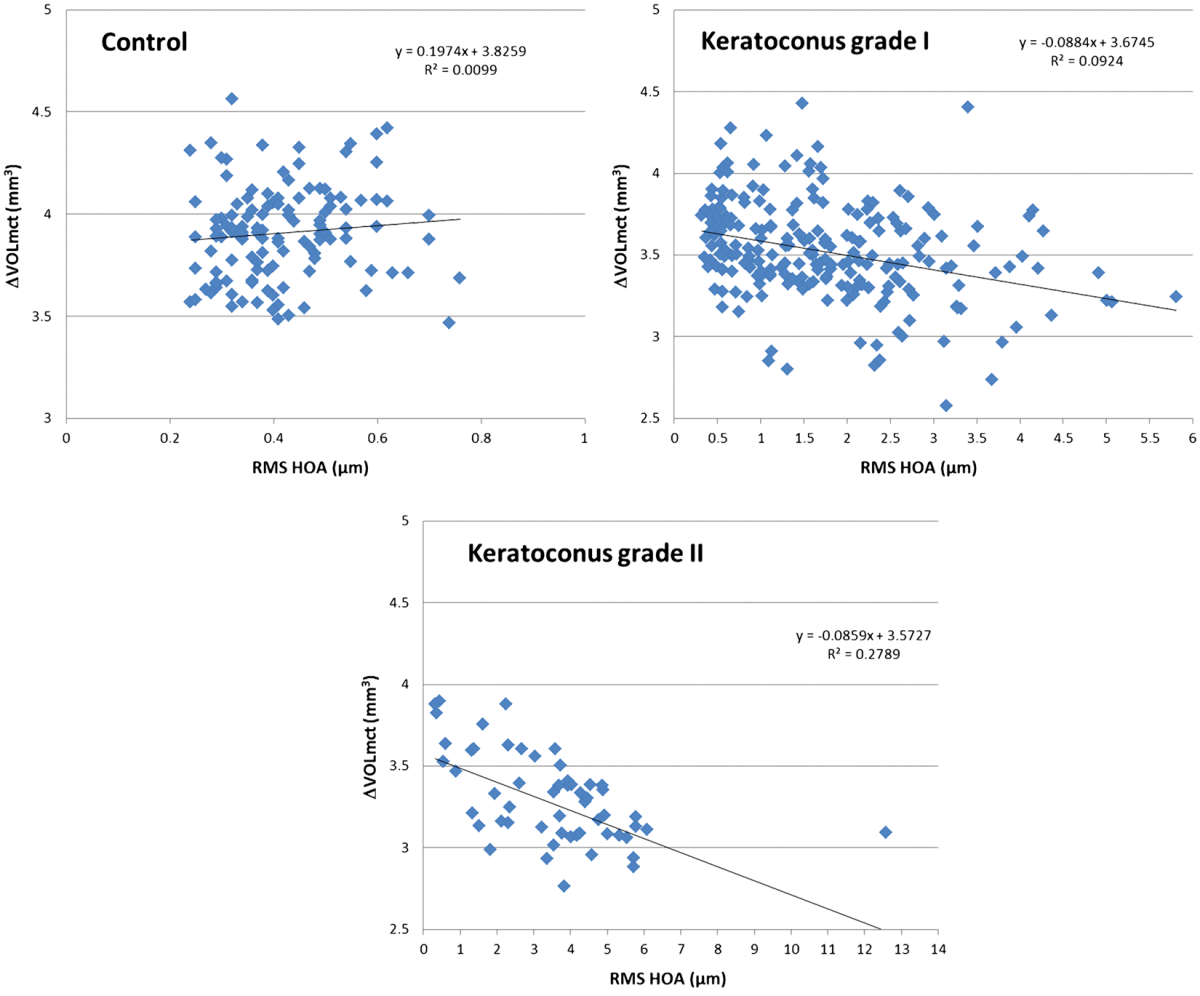
Scatterplots showing the relationship of the change in corneal volume defined by the points of minimal thickness from 0.1 to 1.5 mm of radius for the cylinder of revolution considered for its calculation (ΔVOL_mct_) with the root mean square (RMS) of high order aberration (HOA) in the control (top-left), keratoconus grade I (top-right) and keratoconus grade II groups (bottom). The adjusting line to the data obtained by means of the least-squares fit is shown.

Table 4 summarizes the significant correlations of ΔVOL_mct_, ΔVOL_aap_, and ΔVOL_pap_ with different clinical data in the control group and in the keratoconus subgroups according to the severity stage of the disease. As shown, some statistically significant correlations (although poor) of ΔVOL_mct_, ΔVOL_aap_, and ΔVOL_pap_ with different clinical data were found in the control group. In keratoconus grade I, these corneal volume parameters showed poor (but statistically significant) correlations with corneal aberrometric data (Fig. 3). These correlations between corneal volume parameters and corneal aberrometric data became stronger in keratoconus grade II group (Fig. 3).

**Table 4:** Summary of the correlations obtained between the corneal volume parameters calculated in the control group and keratoconus subgroups according to the stage of severity of the disease.

Coeefficient of correlation (p-value)	Control (C)	Ktc grade I (KC1)	Ktc grade II (KC2)	Ktc grade III (KC3)	Ktc grade IV (KC4)
ΔVOL_mct_ (mm^3^)	Sphere, r = 0.201 (p = 0.025) SE, r = 0.288 (p = = 0.005) Q45, r = −0.247 (p = 0.006) Q8, r = −0.300 (p = 0.001)	Cylinder, r = 0.205 (p = 0.003) CDVA, r = −0.173 (p = 0.012) Total RMS, r = −0.240, (p < 0.001) HOA RMS, r = −0.304, (p < 0.001) Coma RMS, r = −0.297 (p < 0.001) Coma-like RMS, r = −0.289 (p < 0.001) Spherical-like RMS, r = −0.292 (p < 0.001)	Cylinder, r = 0.273 (p = 0.044) Total RMS, r = −0.451, (p < 0.001) HOA RMS, r = −0.528, (p < 0.001) Coma RMS, r = −0.493 (p < 0.001) Coma-like RMS, r = −0.503 (p < 0.001) Spherical-like RMS, r = −0.561 (p < 0.001)	CDVA, r = 0.708 (p = 0.033) SA, r = −0.714 (p = 0.031)	No significant correlations
ΔVOL_aap_ (mm^3^)	Sphere, r = 0.185 (p = 0.039) SE, r = 0.263 (p = 0.011) Q45, r = −0.256 (p = 0.004) Q8, r = −0.335 (p < 0.001)	Cylinder, r = 0.175 (p = 0.010) CDVA, r = −0.154 (p = 0.026) Total RMS, r = −0.200 (p = 0.003) HOA RMS, r = −0.259 (p < 0.001) Coma RMS, r = −0.266 (p < 0.001) Coma-like RMS, r = −0.248 (p < 0.001) Spherical-like RMS, r = −0.223 (p < 0.001)	Total RMS, r = −0.449 (p < 0.001) HOA RMS, r = −0.455 (p < 0.001) Coma RMS, r = −0.429 (p < 0.001) Coma-like RMS, r = −0.441 (p < 0.001) Spherical-like RMS, r = −0.422 (p < 0.001) Q8, r = 0.271 (p = 0.042)	CDVA, r = 0.651 (p = 0.050) SA, r = −0.690 (p = 0.040)	Coma RMS, r = 0.461 (p = 0.047) Coma-like RMS, r = 0.457 (p = 0.049)
ΔVOL_pap_ (mm^3^)	Sphere, r = 0.181 (p = 0.045) SE, r = 0.258 (p = 0.013) Q45, r = −0.255 (p = 0.004) Q8, r = −0.333 (p < 0.001)	Cylinder, r = 0.193 (p = 0.005) CDVA, r = −0.172 (p = 0.013) Total RMS, r = −0.213 (p = 0.001) OA RMS, r = −0.278 (p < 0.001) Coma RMS, r = −0.281 (p < 0.001) Coma-like RMS, r = −0.267 (p < 0.001) Spherical-like RMS, r = −0.245 (p < 0.001)	Total RMS, r = −0.474 (p < 0.001) HOA RMS, r = −0.480 (p < 0.001) Coma RMS, r = −0.457 (p < 0.001) Coma-like RMS, r = −0.465 (p < 0.001) Spherical-like RMS, r = −0.448 (p < 0.001) Q8, r = 0.319 (p = 0.016)	CDVA, r = 0.683 (p = 0.043) SA, r = −0.704 (p = 0.034)	No significant correlations

Abbreviations: UDVA, uncorrected distance visual acuity; SE, spherical equivalent; CDVA, corrected distance visual acuity; Q45 and Q8, corneal asphericity in the central 4.5 and 8 mm; RMS, root mean square; HOA, high order aberrations; SA, spherical aberration; ΔVOL_mct_, change in corneal volume defined by the points of minimal thickness from 0.1 to 1.5 mm of radius for the cylinder of revolution considered for its calculation; ΔVOL_aap_, change in corneal volume defined by the anterior corneal apex from 0.1 to 1.5 mm of radius for the cylinder of revolution considered for its calculation; ΔVOL_pap_, change in corneal volume defined by the posterior corneal apex from 0.1 to 1.5 mm of radius for the cylinder of revolution considered for its calculation.

### Predictive model for incipient keratoconus detection

The logistic regression analysis revealed that the detection of keratoconus grade I was related to the variables: spherical equivalent, Coma RMS, spherical aberration, spherical-like RMS and ΔVOL_aap_ (p > 0.05, Chi-Square and Hosmer-Lemeshow). The coefficient of determination R^2^ Cox and Snell (general) was 0.614, while the R^2^ Nagelkerke (corrected) was 0.862. Table 5 shows the model coefficients (B), the statistical significance, the exponential of B (ExpB, odds ratio) and confidence interval 95% of ExpB for each variable in the model. Specifically, the model revealed that the probability of having keratoconus grade I is 731.93 times higher for each µm increase of Coma RMS, 0.82 times lower for each diopter positive increase of spherical equivalent, 6.12 × 10^27^ times higher for each µm increase of spherical-like RMS, 1.36 × 10^−20^ times lower for each µm increase of primary spherical aberration, and 0.004 times lower for each mm^3^ increase of ΔVOL_aap_. The overall percentage of cases correctly classified by the presented model was 93.4% (90.2% control group, 94.8% keratoconus grade I subgroup), whereas the percentage of cases correctly identified by the classifier of the topography system was 91.6% (96.8% control group, keratoconus grade I subgroup 88.8%). The correlation matrix revealed that weak correlations were present between variables (−0.315 ≤ r ≤ 0.086), except for the relationship between spherical aberration and spherical-like (r = −0.929), inducing an acceptable level of multicollinearity. If spherical aberration variable was eliminated from the model,R^2^ decreased to 0.579. Likewise, a recalculation of the model without including ΔVOL_aap_ showed a decrease in the coefficient of determination R^2^ Cox and Snell to 0.577.

**Table 5:** Summary of model defined for detection of early keratoconus.

	B	Sig	ExpB	CI 95% for ExpB
SE (D)	−0.20	0.015	0.82	0.696 to 0.962
Coma RMS (µm)	6.60	0.004	731.93	8.133 to 6.58 × 10^4^
SA (µm)	−45.74	<0.001	1.36 × 10^−20^	2.15 × 10^−30^ to 8.64 × 10^−11^
Spherical-like RMS (µm)	63.98	<0.001	6.12 × 10^27^	1.31 × 10^16^ to 2.86 × 10^39^
ΔVOL_aap_ (mm^3^)	−5.56	<0.001	0.004	2.64 × 10^−4^ to 0.056
Constant of the model	11.91	0.018	148873.24	—

Abbreviations: SD, standard deviation; Abbreviations: SE, spherical equivalent; RMS, root mean square; SA, spherical aberration; ΔVOL_aap_, change in corneal volume defined by the anterior corneal apex from 0.1 to 1.5 mm of radius for the cylinder of revolution considered for its calculation.

## Discussion

This computational study provides a new insight into the local origin and progression of the complex clinical problem of this rare disease by combining a morphogeometric analysis of the cornea with optical parameters related to the visual quality of patients. In addition, the use of corneal tomography based on the Scheimpflug principle as raw data^17^ source to construct patient-specific three-dimensional geometric models allows this methodology to be susceptible of implementation in commercial devices (interoperability)^18^.

The morphogeometric analysis used in the current study has shown that there are significant differences in volumetric changes in a small area around the points of minimal corneal thickness and the anterior and posterior corneal apex in keratoconus compared to healthy eyes. These results are consistent with those reported by other authors in keratoconus eyes showing a reduction in corneal volume calculated using different approaches^6,7,9,11,12^, all of them characterized by considering the center of reference of the calculated volumes in the geometric center of the cornea. Some authors have demonstrated that there is a significant reduction in corneal volume at 3, 5, 7 and 10 mm in keratoconus eyes compared to controls^6,11^. Ahmadi Hosseini *et al*.^7^ demonstrated that this reduction in corneal volume was related to a lower percentage thickness increase in keratoconus. Ambrósio *et al*.^8^ calculated in a sample of keratoconus and healthy eyes the corneal volume within diameters from 1.0 to 7.0 mm with 0.5 mm steps centered on the thinnest point to create a corneal-volume distribution. With this analysis, they found significant differences between healthy and keratoconus eyes in the corneal-volume distribution as well as in the percentage increase in volume between 3.5 and 7.0 mm^8^.

In this study, the analysis of corneal volume has been focused using as a reference center four points that are normally altered in keratoconus: the points of minimal corneal thickness^19^ and the anterior and posterior corneal apexes^9,20–23^. It should be considered that the affected area in the posterior surface of the keratoconic cornea has been found to be located at about 1.5–2 mm from the corneal center on the 135 degrees hemimeridian^9^. Small variations in the radius of the cylinder of revolution used for corneal volume calculations lead to significant differences between healthy and keratoconus eyes. Therefore, the corneal volume approach presented in this study seems to be useful for differentiating between healthy and keratoconus corneas. To our knowledge, this is the first time that corneal volume calculations centered on anterior and posterior corneal apex in keratoconus are reported, which shows the same trends than those centered on the points of minimal thickness. There is a minimal difference between VOL_mct_ and VOL_aap_ or VOL_pap_, with a trend to lower values for VOL_mct_. Auffarth *et al*.^21^ demonstrated that there was a separation in keratoconus corneas between the apexes and the points of minimal corneal thickness that may explain the minimal difference between our corneal volume parameters. Specifically, they found a mean value for this distance of 0.917 ± 0.729 mm^21^.

Besides the discrimination between healthy and keratoconus groups, significant differences were found in the change of VOL_mct_, VOL_aap_ and VL_pap_ from 0.1 to 1.5 mm of radius for the cylinder of revolution considered for their calculation between keratoconus severity subgroups, except among the two most advanced stages (grade III and IV). This is also consistent with the results of Mannion and colleagues^11^, reporting a decrease in the corneal volume estimation provided by the Pentacam system from Oculus in moderate and severe keratoconus. These authors suggested that this might be in relation to the loss of corneal tissue due to the progressive degeneration of the corneal structure. Indeed, Ahmadi Hosseini *et al*.^6^ confirmed that reduction of both corneal volume and biomechanical properties (measured with the Ocular Response Analyzer from Reichert) occurred in keratoconus.

In spite of all this previous experience showing the reduction of corneal volume in keratoconus, there are no studies reporting a moderate predictive ability of this parameter for detecting this pathological condition^6^. Likewise, although topographic, pachymetric and aberrometric parameters have been shown to be useful for keratoconus detection^4^, they are not sensible enough for detecting early central keratoconus^24^. Indeed, it has been demonstrated that more stringent diagnostic criteria are required for topographic and pachymetric criteria to avoid missing pathological cases, but with the consequent reduction in specificity^24^. Considering that the correlation between corneal volume changes and aberrations increased with increase in the severity of the disease, we decided to obtain a predicting model by logistic regression for the detection of incipient keratoconus (grade I) based on the combination of corneal volume and corneal aberrometric data. It should be considered that corneal aberrations are correlated with the severity of the disease^25^ and is even used as criteria for grading keratoconus^26^. This predicting model has been performed only considering keratoconus grade I, as moderate and advanced keratoconus can be easily detected by means of conventional topographic analyses and the real challenge is to detect with accuracy those cases of keratoconus in an incipient stage. We found that considering refraction or second order aberrations (spherical equivalent), corneal high order aberrations (spherical, coma and spherical-like aberrations) and ΔVOL_aap,_ the detection of keratoconus grade I could be done correctly in 94.7% of cases. In contrast, the classifier of the topography system used only detected correctly 88.8% of cases. This system has been reported to provide true predictions in around 93% of cases or more^16^. It should be considered that this system uses anterior and posterior topographic asymmetries, corneal aberrometric data and the points of minimal corneal thickness to perform its predictions^16^. Concerning the sample used, the inclusion of a significant sample of central keratoconus may have led to some missing pathological cases as they do not present geometric symmetries and high levels of coma aberration^24^. For this reason, the inclusion of the reduction of the corneal volume should be a crucial factor to be included in predictive models, as demonstrated in the current study. Finally, it should be remarked that the levels of sensitivity and specificity associated to the presented model (94.8% and 90.2%) are equivalent and even better than those reported by other different models of keratoconus detection based on standard topographic analysis^16,27–30^.

In conclusion, there is a clear reduction of corneal volume in early keratoconus, and such reduction increases significantly with the severity level of the disease according to the Amsler-Krumeich grading system. The combination of optical and volumetric data calculated considering the anterior corneal apex as a reference point allows the clinician to perform an accurate detection of incipient cases.

In future works, some of the present study’s limitations will be resolved, as for instance:

- The requirement of using only raw valid data from the corneal topographer to ensure obtaining an accurate 3D model of the cornea led to discard data from radii 4.2 mm to 6 mm. With this regard, a point cloud reconstruction algorithm or method should be developed in the future, avoiding to loose these captured data.
- The filtering process applied to all CSV files from the Sirius device in order to eliminate those cases with erroneous data within radii from 0 mm to 4 mm led to a reduction in the data sample of the observational comparative study.
